# Use of machine learning to improve the estimation of conductivity and permittivity based on longitudinal relaxation time T1 in magnetic resonance at 7 T

**DOI:** 10.1038/s41598-023-35104-9

**Published:** 2023-05-15

**Authors:** Daniel Hernandez, Kyoung-Nam Kim

**Affiliations:** 1 Neuroscience Research Institute, Gachon University, Incheon, 21988 Korea; 2 Department of Biomedical Engineering, Gachon University, Seongnam, 13120 Korea

**Keywords:** Biomedical engineering, Preclinical research, Biomarkers

## Abstract

Electrical property tomography (EPT) is a noninvasive method that uses magnetic resonance imaging (MRI) to estimate the conductivity and permittivity of tissues, and hence, can be used as a biomarker. One branch of EPT is based on the correlation of water and relaxation time T1 with the conductivity and permittivity of tissues. This correlation was applied to a curve-fitting function to estimate electrical properties, it was found to have a high correlation between permittivity and T1 however the computation of conductivity based on T1 requires to estimate the water content. In this study, we developed multiple phantoms with several ingredients that modify the conductivity and permittivity and explored the use of machine learning algorithms to have a direct estimation of conductivity and permittivity based on MR images and the relaxation time T1. To train the algorithms, each phantom was measured using a dielectric measurement device to acquire the true conductivity and permittivity. MR images were taken for each phantom, and the T1 values were measured. Then, the acquired data were tested using curve fitting, regression learning, and neural fit models to estimate the conductivity and permittivity values based on the T1 values. In particular, the regression learning algorithm based on Gaussian process regression showed high accuracy with a coefficient of determination R^2^ of 0.96 and 0.99 for permittivity and conductivity, respectively. The estimation of permittivity using regression learning demonstrated a lower mean error of 0.66% compared to the curve fitting method, which resulted in a mean error of 3.6%. The estimation of conductivity also showed that the regression learning approach had a lower mean error of 0.49%, whereas the curve fitting method resulted in a mean error of 6%. The findings suggest that utilizing regression learning models, specifically Gaussian process regression, can result in more accurate estimations for both permittivity and conductivity compared to other methods.

## Introduction

Magnetic resonance imaging (MRI) is a useful modality for clinical practices for the non-invasive diagnosis of tumors, injuries, and the overall functional state of the human body^1–6^. MR images are acquired based on the signals provided by the resonance of protons; in the human body, protons are linked to water molecules and allow signal acquisition of the anatomy. The characterization and modeling of tissue properties are crucial for the development of new diagnostic and therapeutic strategies. Each tissue has its own intrinsic properties, such as relaxation times T1 and T2; therefore, it is possible to distinguish between organs and tumors^7–10^. Similarly, each tissue has specific electrical properties (EP), in terms of conductivity (σ) and permittivity (ε)^11–13^. Electrical property tomography (EPT) is used to analyze the computation of EP in a non-invasive manner using MRI protocols^14–16^. Special interest has been given in the use of conductivity as a biomarker for detecting and studying the evolution of tumors^15,17,18^.

The standard method for performing EPT includes acquiring images related to the phase of the transmitted magnetic |B1| field^16^. Conductivity can be obtained based on the approximation of the solution to the Helmholtz equation^16,19^. However, this method has its limitation considering it is based on a double derivative or Laplacian, which produces noise and discontinuity^14,16^. However, several methods have been proposed to minimize the noise and fix this artifact using image filters and algorithms to compute the derivates^20–22^.

To circumvent the use of derivatives and image filters, a study proposed a method to estimate conductivity and permittivity based on the water content of the tissue^23^. According to the Maxwell mixture theorem^24^, electrical properties are proportional to the portion of water considering the tissues in the human body have large concentrations of water in different proportions^25–27^, and MRI acquisition is primarily based on the signal derived from the protons in the water molecules. The water content was estimated using T1-weighted images. In the original work, two curve-fitting functions were proposed to correlate the water content with permittivity and conductivity^23^. The results provided an image quality similar to that of the anatomical high-resolution MR image without noise amplification or filter effects. However, only a few tissues and water concentrations were considered, which limit the generalization of the model, as tissues with different electrical properties may not fall into the model.

A recent study evaluated the correlation between the relaxation time T1 and EP without considering the water content^28^. T1 is the relaxation time, which indicates that the time spins return to the equilibrium state after excitation with an external magnetic field. T1 is used as a biomarker to identify tissues because its value depends on the type of molecular structure and the surrounding environment. In^28^, a general formulation was proposed to understand the relationship between T1 and EP, at least for a phantom setup. The results showed that a high correlation existed between T1 and permittivity, which follows a formulation similar to Debye’s equations for the relaxation times of electrical permittivity^29,30^. Similarly, a formulation was proposed to estimate conductivity based on permittivity and T1^28^. Although the experiments followed the hypothesis and formulation, the conductivity estimation was dependent on the concentration of sodium or any substance that could modify the conductivity. Therefore, this study uses machine learning and artificial intelligence to predict and compute conductivity values from a given dataset.

Improvements in the above-mentioned EPT methods could benefit from the use of machine learning (ML) or artificial intelligence (AI) methods. The use of ML and AI for biomedical imaging has been proposed for several areas of MRI^31–33^, such as image segmentation, image registration, and filtering^34,35^. Notably, the use of AI for EPT has also been proposed to solve the problem of noise produced using the derivatives of the phase maps used to compute conductivity^36–38^. Similarly, a concept based on image intensity and deep learning was proposed to estimate conductivity^39^.

Regression learning (RL) algorithms are machine learning methods^40^ wherein the algorithm is trained to learn the relationships in the given data. RL is a supervised model, and for this model, the training data must be labeled. Some common algorithms for regression learning are linear regression^41^, regression trees^42^, support vector machines (SVM)^43^, and Gaussian process regression (GPR)^44^.

The popularity of neural network fit (NNF) has increased in recent years; in particular, an algorithm has been developed using MATLAB via neural network fitting^45^. The fitting problems were solved using a two-layer feed-forward network. The Levenberg–Marquardt algorithm, Bayesian regularization, and a scaled conjugate gradient were used in the development of the aforementioned algorithm.

This study presents promising results with significant potential for clinical applications, as the acquired models can be used to determine the conductivity and permittivity of tissues. By establishing a relationship between tumor tissue T1 and conductivity, this approach can aid in the understanding of tumor properties and potentially be used as biomarker for diagnosis and monitoring of tumors. This work emphasizes the importance of accurate modeling and characterization of phantom conductivity and permittivity based on T1 values, such that similar methodology can be applied for tissue properties and clinical research. This study improves the estimation accuracy reducing the square root mean error of conductivity by 14% and permittivity by 18% based on the relaxation time T1 by using ML and AI methods, when compared to the curve fitting method. ML methods are based on regression learning, whereas the AI is based on neural network fitting. We developed multiple imaging samples with a unique mixture of ingredients to modify the conductivity and permittivity. To train the algorithms, each sample was measured using a dielectric assessment kit. This measurement is invasive because the probe must be in contact with or submerged in the mixture. However, the goal is to measure the EP values in a non-invasive manner using MRI.

## Methods

### Overall

**Figure 1:**
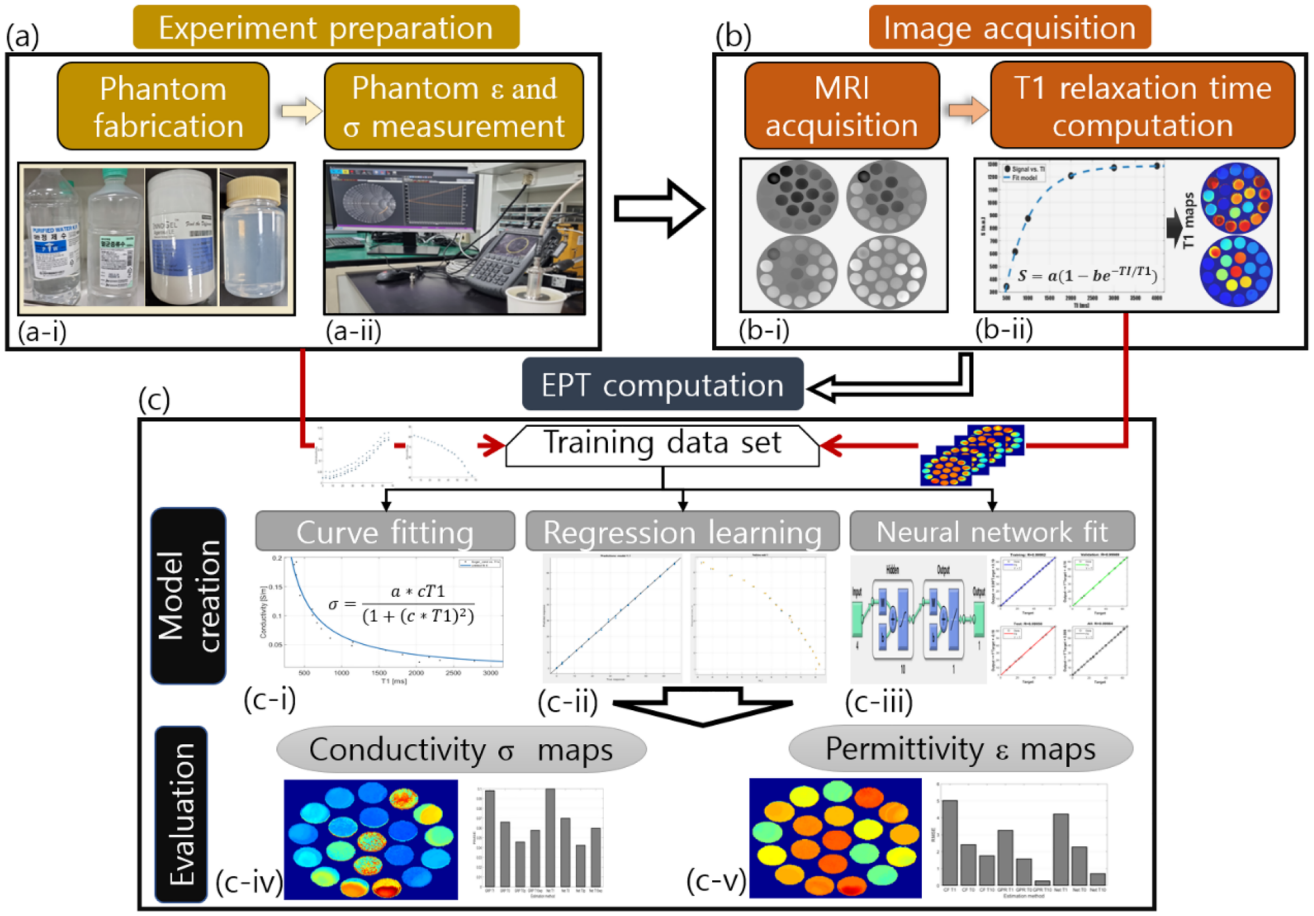
Diagram flow of the present work (**a**) experiment preparation comprising (**a**-i) phantom fabrication and (a-ii) and dielectric measurements; (**b**) image acquisition with MRI (**b**-i) and (**b**-ii) estimation of T1 values. The data gathered with (**a**-ii) and (**b**-ii) are used for EPT computation; (**c**) model creation with (**c**-i) curve fitting, (**c**-ii) regression learning, and (**c**-iii) neural network fit. The output is the (**c**-iv) permittivity and (**c**-v) conductivity maps.

The study methodology can be divided into three parts: (1) experimental preparation, (2) image acquisition, and (3) EPT computation. Figure 1 shows the experimental procedure. The experiment (Fig. 1a) comprised of making of the phantoms (Fig. 1a-i). For each phantom, we measured the permittivity and conductivity (Fig. 1a-ii) using an invasive method, which provided the true values that were then used for training and validation. We acquired the MR images for each fabricated phantom (Fig. 1b-i) and computed the T1 relaxation times (Fig. 1b-ii). A dataset was created by combining the measured data (conductivity and permittivity) and the relaxation time T1 that corresponded to each phantom; the developed dataset was then used for training, evaluation, and validation. For the computation of the EPT, we used three methods: conventional curve fitting, regression learning algorithms, and neural network fitting algorithms. Curve fitting was performed based on a previously published formulation, and this method was used as a comparison parameter. Conductivity and permittivity maps were created based on the models acquired using RL and NNF. A detailed description of each step is provided as follows.

### Phantom construction

**Figure 2:**
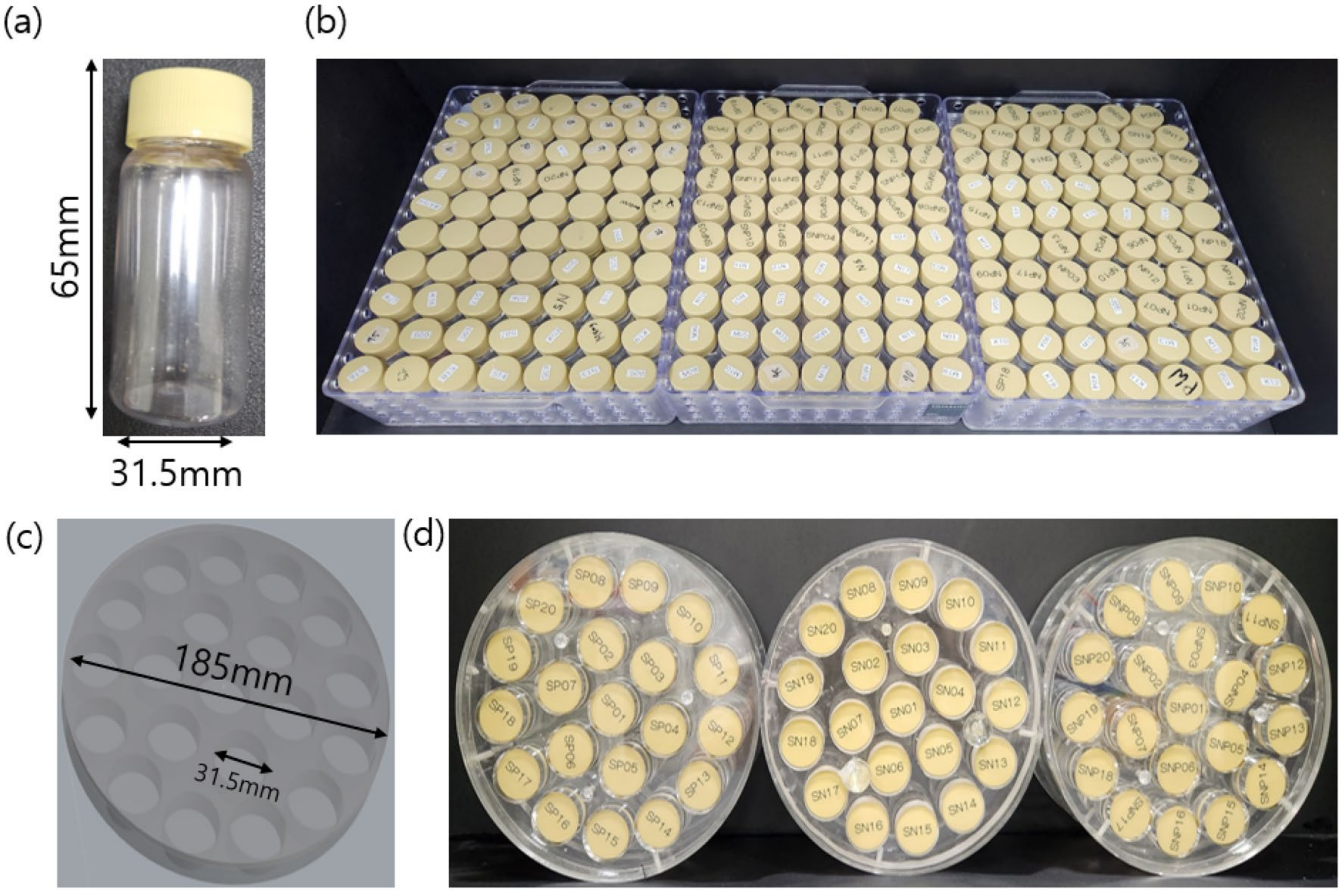
Picture of a single phantom (**a**, **b**) the total phantoms made. The dimensions of the (**c**) acryl for placing the phantoms and (**d**) the final set up used for MR imaging.

In total, 140 phantoms were prepared, each comprising a mixture of water, sucrose, sodium, and potassium at different concentrations. The mixtures were placed in 65 mm long cylindrical containers with a diameter of 31.5 mm and volume of 50 ml, as shown in Fig. 2a. Figure 2b shows all the phantoms made. To acquire the MR image simultaneously, we created an acryl frame such that the phantoms could be placed and fixed inside the MRI scanner. Figure 2c shows a 3D model of the acryl case, which comprises 20 holes with a diameter of 31.5 mm and a height of 80 mm, and a total diameter of 185 mm. The cover of the container could tighten and fix the phantoms. This setup allows the acquisition of 20 phantoms for each MR scan, which saves time, and allows the interchange of phantoms, as needed, as shown in Fig. 2d.

**Figure 3:**
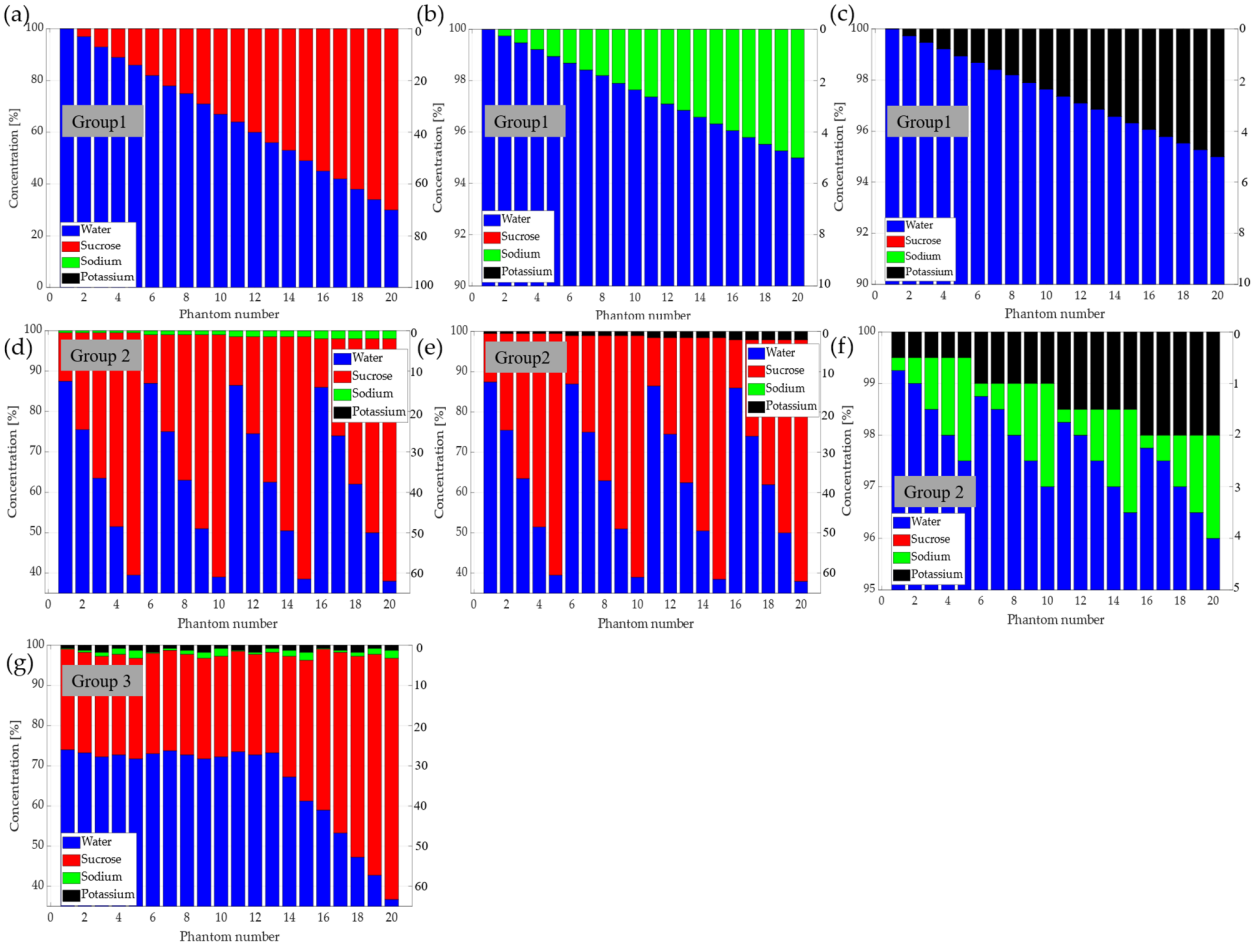
The graphical representation of the ingredients used for each phantom, with mixture of group 1: (**a**) water-sucrose, (**b**) water-sodium, (**c**) water-potassium, group 2: (**d**) water-sucrose–sodium, (**e**) water-sucrose-potassium, (**f**) water-sodium–potassium and group 3: (**g**) water-sucrose–sodium-potassium.

The phantoms were divided into three groups depending on the number of ingredients used. The first group comprised a single water mixture, as follows: (1) 20 phantoms containing water and sucrose, where the sucrose percentage (red bars) was controlled between 0 and 70% in steps of 4%, as shown in Fig. 3a. (2) 20 phantoms containing water and sodium (NaCl), as shown in Fig. 3b. 0 to 5% sodium was added in steps of 0.25%, and (3) 20 phantoms containing water and potassium at concentrations ranging from 0 to 5% was added in steps of 0.25%, as shown in Fig. 3c. The second group comprised double mixtures. (4) 20 phantoms of a mixture of water, sucrose, and sodium, as shown in Fig. 3d, where sucrose was added from 12 to 60% in steps of 12% for each concentration of sodium from 0.5 to 2% in steps of 5%; (5) similarly, 20 phantoms of water, sucrose, and potassium (Fig. 3e) were prepared with sucrose concentrations ranging from 12 to 60% in steps of 12% for each potassium concentration ranging from 0.5 to 2% in steps of 0.5%; (6) 20 phantoms with a mixture of water, sodium, and potassium, as shown in Fig. 3f, with sodium concentrations of 0.25, 0.5, 2, 1.5%, and 2%, and potassium concentrations ranging from 0.5 to 2% in steps of 0.5%; finally, (7) the third group comprised a mixture of water, sucrose, sodium, and potassium, as shown in Fig. 3g. In a total of 20 phantoms, 13 phantoms had a concentration of 25% sucrose and 7 phantoms had concentrations ranging from 30 to 60% in steps of 5%, whereas sodium was added in concentrations between 0.5% and 2.4% and potassium with concentrations between 0.75 and 1.75% in steps of 0.75%.

The phantoms were prepared and maintained at 20 °C, and special care was taken to avoid air bubbles. The selection of the concentration of the ingredients was based on a range of values that could help to understand the correlation between each ingredient alone and in combination with other ingredients.

### Dielectric measurement

The true conductivity and permittivity of each phantom were measured using a Dielectric Assessment Kit (DAK model 12, SPEAG, Zurich, Switzerland). The data were acquired in the frequency bandwidth ranging from 200 to 400 MHz. To expand the dataset, the measurements were repeated thrice for each phantom. The total dataset comprised three measured data points and the average of the three measurements. The measured matrix was 140 × 2 × 4 for 140 total phantoms, conductivity, permittivity, and four measurements.

### MRI experiments

The images were acquired with each phantom using a 7 T MRI scanner (Magnetom, Siemens AG, Berlin, Germany). Turbo inverse recovery images were acquired for each phantom, with a TI of 200, 500, 700, 1000, 2000, 3000, and 4000 ms and a TR of 12,000 ms. The matrix size of each image was 320 × 320 × 3.

### T1 value measurement

Using the inverse recovery images, T1 values were computed as:

1 $$S=a(1-b {e}^{-TI/T1})$$

where *S* is a vector of pixel values of the images acquired with each inversion time TI, and the coefficients $a$, $b$, and T1 are determined by performing curve fitting with a nonlinear least-squares method. Coefficient $a$ corresponds to the proton density and coefficient $b$ is related to the ratio of the flip angle (FA). When the applied flip angle was 90°, the coefficient $b$ was 2. However, due to the nature of ultrahigh frequencies that are used in 7 T MRI, the delivered flip angle is not uniform across all imaging areas, resulting in image intensity variations. Therefore, it is important to correct T1 maps based on the RF pulse efficiency^46^. In this work, we use the following normalization: The T1 maps are scaled by the logarithm of the coefficient b, given as:

2 $$T0=T1 \mathrm{log}(b)$$

Additionally, the scaled T0 value corresponds to the time at which the value of S in (1) is equal to zero, that is, the time at which the magnetization is zero. The value of T0 results in a more uniform distribution while maintaining a correlation with T1.

### Estimation of conductivity and permittivity based on curve fitting

Based on the correlation^28^, permittivity ε can be expressed as a function of T1 or T0 as:

3 $$\varepsilon ={e}_{1}+\frac{{e}_{2}-{e}_{1}}{1+{(c T0)}^{2}}$$

The coefficients ${e}_{1}$, ${e}_{2}$ and $c$ are obtained after performing curve fitting of Eq. (3) using a nonlinear least-squares method. Similarly, the conductivity can be determined using the equation proposed in previous work^28^, which reported the relation between T1 and loss index *ε*″. The equations can be updated for conductivity σ using the following relationship:

4 $$\sigma = w\varepsilon_{0} \varepsilon \prime$$

where $w$ is the angular frequency and ε_0_ is the permittivity of the free space. The equation used for conductivity is given as:

5 $$\sigma =\frac{w{\varepsilon }_{0}{s}_{1}fT0}{1+{({s}_{2}fT0)}^{2}}$$

where the coefficients s_1_, s_2,_ and *f* are determined using the curve fitting procedure based on the nonlinear least square method. The value of *f* reportedly represents the percentage of sodium in the mixture. Furthermore, a second equation was proposed based on the complex geometric relationship between conductivity and permittivity, which is also scaled by the sodium percentage as:

6 $$\sigma =(w{\varepsilon }_{0}\sqrt{{k}^{2}-{(\varepsilon -cr)}^{2}})sc$$

where *k* is the radius of the circumference that relates the conductivity and permittivity, *cr* is the center of the semicircle, and *sc* is a scaling factor that represents the percentage of sodium in the mixture, which varies from 0 to 1.

### Dataset

The dataset for the machine learning and neural network algorithms was created from the measured electrical properties of each phantom and the values of T0 acquired with MR images. T0 for each phantom was extracted from the T0 maps by averaging a region of interest (ROI) of 20 × 20 pixels at the location of each phantom.

The dataset comprised 140 phantoms, two variables (permittivity and conductivity), T0 values corresponding to four slices of images, and four measurements with the DAK system. The total matrix size was 140 × 2 × 4 × 4. This dataset was used for the training, evaluation, and validation. For each phantom, the total matrix of this study can be described as:

7 $${\text{D}} = \{ {\text{water}},{\text{ sucrose}},{\text{ sodium}},{\text{ potassium}},\;\sigma ,\varepsilon ,{\text{ T1}},{\text{ T}}0\} ,$$

However, two separate models were created: one for permittivity and another for conductivity. The permittivity model had a single input, T0, and the development was performed using the matrix:

8 $${\text{De}} = \left\{ {\varepsilon ,{\text{ T}}0} \right\},$$

Three models for conductivity were tested: for phantoms containing sodium (Ds1), for phantoms with potassium (Ds2), and all combined together (Ds3). The models had a double-input variable T0 and permittivity, such as the matrix for creating the model.

9 $${\text{Ds1}} = \{ {\upsigma },{\upvarepsilon },{\text{ T}}0\} /{\text{sodium}},$$

10 $${\text{Ds2}} = \{ {\upsigma },{\upvarepsilon },{\text{ T}}0\} /{\text{potassium}},$$

11 $${\text{Ds3}} = \left\{ {{\upsigma },{\upvarepsilon },{\text{ T}}0} \right\}/{\text{all}},$$

Model design, training, and evaluation were performed using MATLAB 2018 (MathWorks, Inc., Natick, Massachusetts, United States.) with a Regression Learner and Neural Network Fitting apps included in the machine learning toolbox. The performance of each algorithm was evaluated using the root mean square error (RMSE) and coefficient of determination R^2^ value.

### Regression learning algorithms

The regression learner algorithms were categorized into linear regression, regression tree, support vector machine, ensemble, and Gaussian regression processes. A cross-validation setting was used to avoid overfitting, and a value of 5 was selected. 20 algorithms based on RL were tested, and Table 1 shows the names of the algorithms and model types.

**Table 1:** Performance of regression learning algorithms for permittivity estimation.

Algorithm name	Model type	RMSE	R^2^
Linear regression	Linear	5.11	0.75
Linear regression	Interactions linear	5.11	0.75
Linear regression	Robust linear	5.25	0.73
Linear regression	Stepwise linear	5.11	0.75
Tree	Fine tree	2.32	0.95
Tree	Medium tree	2.50	0.94
Tree	Coarse tree	2.87	0.92
SVM	Linear SVM	5.47	0.71
SVM	Quadratic SVM	19.3	– 2.64
SVM	Cubic SVM	56.53	– 30.53
SVM	Fine Gaussian SVM	3.16	0.90
SVM	Medium Gaussian SVM	3.49	0.88
SVM	Coarse Gaussian SVM	4.63	0.79
Ensemble	Boosted trees	3.78	0.86
Ensemble	Bagged trees	2.32	0.95
GPR	Squared exponential GPR	2.74	0.93
GPR	Matern 5/2 GPR	2.70	0.93
GPR	Rational quadratic GPR	2.02	0.96
GPR	Exponential GPR	1.90	0.96

### Neural network fitting

The neural network fit was tested using Levenberg–Marquardt^47–49^, scaled conjugate gradient^50^, and Bayesian regularization^51^, provided by the toolbox. The Levenberg–Marquardt algorithm is designed to approximate the Hessian matrix by implementing a Jacobian matrix in order to speed up the training. The scaled conjugate gradient algorithm is based on gradient search, and the Bayesian regularization minimizes a linear combination of square errors and weights. The data were divided into 70% for training, 15% for validation, and 15% for testing. The neural network comprised an input, a hidden layer, an output layer, and an output. The network size was adjusted, as described in Tables 2 and 4.

**Table 2:** The performance of Neural fitting algorithms for permittivity estimation.

Algorithm name	Network size	Results	RMSE	R^2^
Levenberg–Marquardt	17	Training	2.6	0.93
Levenberg–Marquardt	17	Validation	2.8	0.91
Levenberg–Marquardt	17	Testing	2.7	0.92
Levenberg–Marquardt	10	Training	2.9	0.91
Levenberg–Marquardt	10	Validation	3.1	0.89
Levenberg–Marquardt	10	Testing	3.5	0.90
Levenberg–Marquardt	50	Training	2.4	0.94
Levenberg–Marquardt	50	Validation	2.2	0.91
Levenberg–Marquardt	50	Testing	2.4	0.95
Bayesian regularization	10	Training	2.7	0.92
Bayesian regularization	10	Validation	2.5	0.92
Bayesian regularization	10	Testing	2.6	0.92
Scaled conjugate gradient	10	Training	3.0	0.90
Scaled conjugate gradient	10	Validation	2.7	0.84
Scaled conjugate gradient	10	Testing	3.1	0.89

## Results

### Dataset

**Figure 4:**
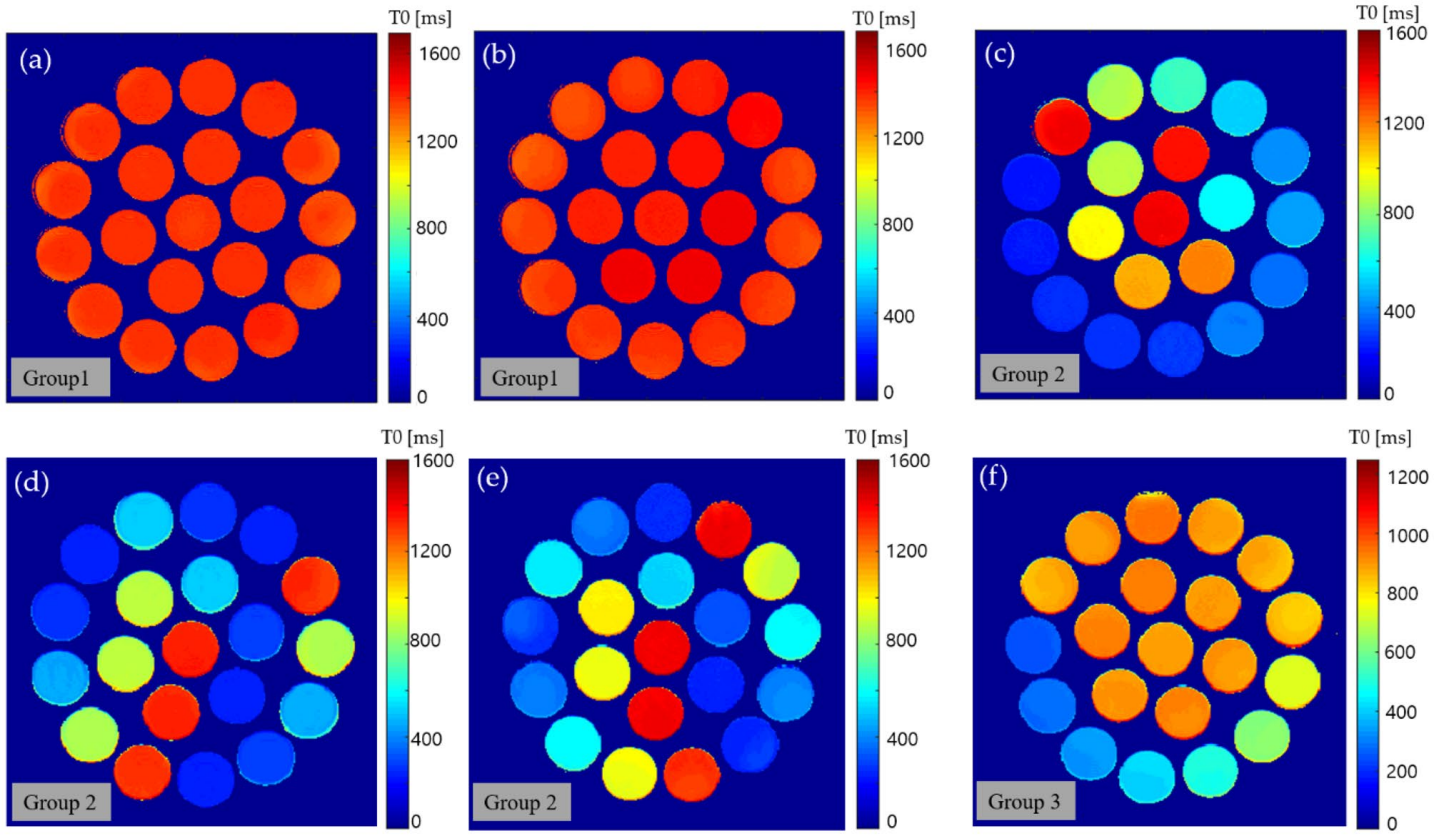
The computed T0 maps for each of the phantom mixtures of group 1: (**a**) water-sodium, (**b**) water-potassium, (**c**) water-sucrose, group 2: (**d**) water-sucrose–sodium, (**e**) water-sucrose-potassium, and group 3: (**f**) water-sucrose–sodium-potassium.

The dataset comprised the electrical properties and T1 values of each phantom. The electrical properties were measured using DAK, for which the values of the respective conductivity and permittivity were measured. The T1 values and normalized T0 values were computed using Eqs. (1) and (2), respectively. Figure 4 shows the computed T0 maps for each phantom type. The T0 map for the mixture of water and sodium, and water and potassium are plotted in Fig. 4a and b, respectively. From these maps, it can be seen that the T0 value is rather uniform, and the value does not vary significantly between phantoms ranging from 1400–1500 ms; however, in the case of T1, the range was between 1500 and 3600 ms. Figure 4c shows the T0 maps from the mixture of water and sucrose and shows the change in T0 based on the concentration of sucrose. Figure 4d,e plots the T0 maps for the phantom mixtures of water-sucrose–sodium and water-sucrose-potassium, respectively, which show the contrast variation for each phantom. The T0 map for the phantoms with a mixture of water, sucrose, and sodium potassium is shown in Fig. 4f. A summary of the average values of the measured conductivity, permittivity, T1, and T0 for each phantom is included in supplementary data.

**Figure 5:**
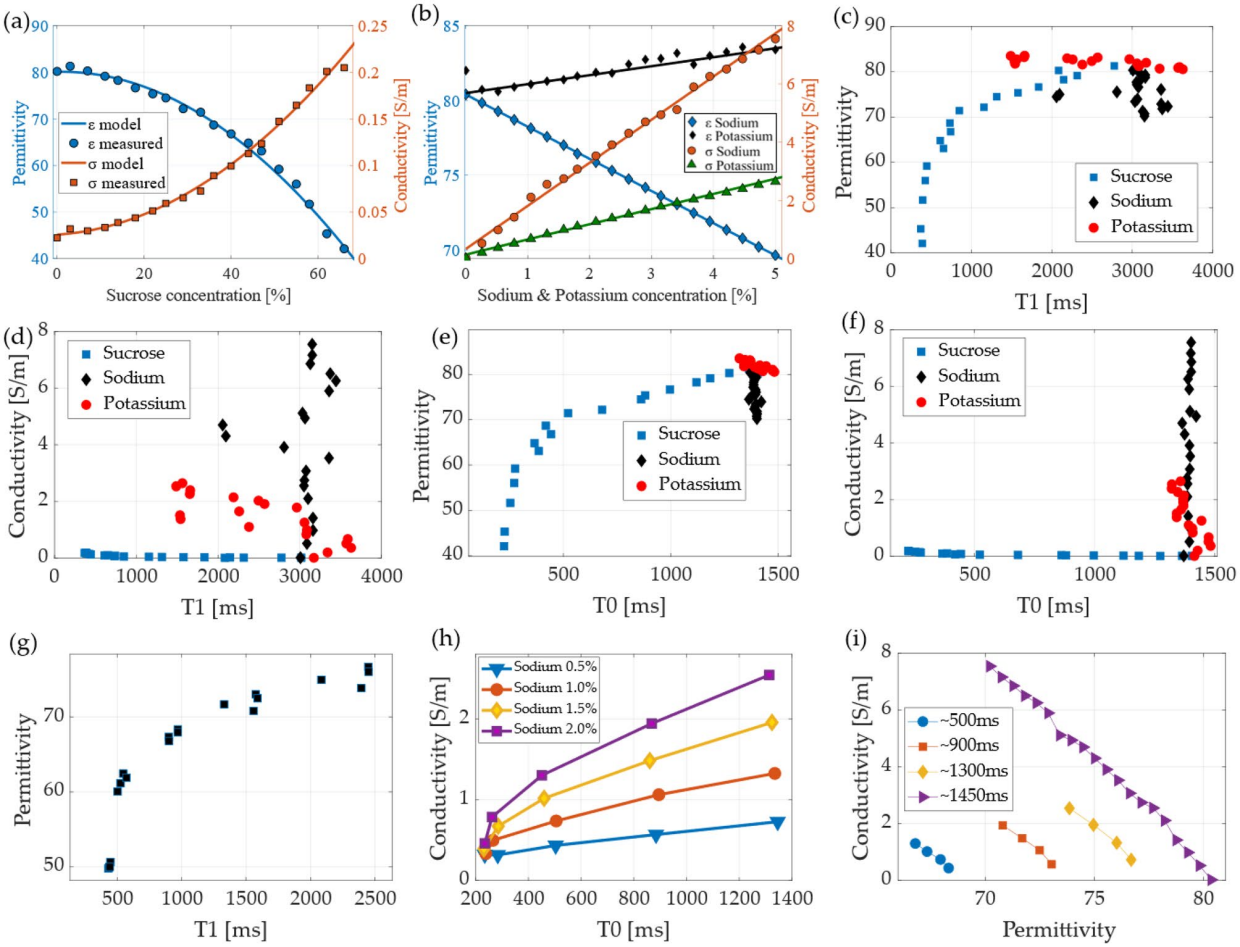
The plots of the measured dielectric properties: permittivity and conductivity. (**a**) In relation to the sucrose concentration for the water-sucrose mixtures and (**b**) in relation to the sodium and potassium for the water-sodium and water-potassium mixtures, respectively. The relationship between (**c**) T1 and permittivity indicates the mixtures water-sucrose, water-sodium, and water-potassium; (**d**) the relationship between T1 and conductivity for the mixtures water-sucrose, water-sodium, and water-potassium; (**e**) the plot of T0 and permittivity; (**f**) the plot of conductivity and T0; (**g**) the T1 and permittivity for the water-sucrose–sodium mixture; (**h**) T0 and conductivity organized by the concentration of sodium for the water-sucrose–sodium mixture; and (**i**) the relationship between permittivity and conductivity arranged by the T0 values.

Figure 5 plots a summary of the correlation between the input variables, arranged by ingredient concentration, T1, T0, permittivity, and conductivity. Figure 5a shows a plot of the phantoms that were made using only water and sucrose and shows the variation of permittivity and conductivity depending on the sucrose concentration. The sucrose concentration causes the permittivity to vary between 80 and 40, which can be modeled as a second order polynomial equation with a R^2^ of 0.99 and RMSE of 0.99, as indicated in Eq. (11), where S represents the percentage [%] of sucrose. The relation with the conductivity can also be modeled with a second order polynomial (12) with a fit of R^2^ 0.98 and RMSE of 0.0065, the measurements indicate that sucrose change the conductivity in a range from 0.01 to 0.2 S/m.

12 $${\upvarepsilon }_{{({\text{water}} - {\text{sucrose}})}} = - 0.00{\text{88 S}}^{{2}} + 0.{\text{169 S}} + {8}0.0{9},$$

13 $${\upsigma }_{{({\text{water}} - {\text{sucrose}})}} = {4}.{\text{11e}} - {\text{5 S}}^{{2}} + 0.000{\text{129 S}} + 0.0{22}.$$

In Fig. 5b, the concentration of sodium and potassium is plotted against the permittivity and conductivity of the water-sodium and water-potassium phantoms. It can be seen that the permittivity change in relation to potassium concentration is small and uptrend; however, the permittivity change due to sodium displays a negative linear relation. The permittivity of the sodium and potassium can be modeled as a linear equation as:

14 $${\upvarepsilon }_{{({\text{water}} - {\text{sodium}})}} = - {2}.{\text{15 Na}} + {8}0.{39},$$

15 $${\upvarepsilon }_{{({\text{water}} - {\text{potassium}})}} = 0.{\text{59 K}} + {8}0.{49},$$

with Na and K representing the percentage of sodium and potassium, respectively. The fitting models had a R^2^ of 0.98 and 0.94 for the (13) and (14), respectively.

A positive linear relationship can be seen in the case of conductivity when compared to the concentration of sodium and potassium^28,52^. The sodium concentration exhibits a higher slope of conductivity compared to potassium. The measured data can be fit by using a linear model as:

16 $${\upsigma }_{{({\text{water}} - {\text{sodium}})}} = {1}.{\text{48 Na}} + 0.{33},$$

17 $${\upsigma }_{{({\text{water}} - {\text{potassium}})}} = 0.{\text{52 K}} + 0.{13},$$

both fitting models had a R^2^ of 0.99. For the mixture with only water, a concentration of 5% sodium has a conductivity of 8 S/m; however, in the case of human tissues, the higher value of conductivity that has been reported is in the CSF and is approximately 2.2 S/m^12,13^, which corresponds to approximately 1.8% of sodium for the case of the water-sodium mixture. The plots of the EP values from the single mixture phantoms help us understand the relationship and correlation. Figure 5c plots the T1 and permittivity for the phantoms of a single mixture, water-sucrose, water-sodium, and water-potassium labeled in the plot as sucrose, sodium, and potassium, respectively. This plot also illustrates the correlation between permittivity and T1, as described in^28^. Figure 5d plots T1 and conductivity for the single mixture phantoms of sucrose, sodium, and potassium. A better description of the relationship can be achieved when using T0, as in Figs. 5e and f for permittivity and conductivity, respectively. The use of T0 shows a less dispersed data, especially in the case of conductivity, where the concentration of sodium appears as a vertical line, which is in accordance with Eq. (5). A similar pattern can be seen for the case of the double mixed phantoms (water-sucrose–sodium), which are plotted in Figs. 5g and h for the permittivity based on T1 and conductivity based on T0, respectively. The plot in Fig. 5h is of particular interest as it shows that the curve of T0 vs conductivity also depends on the concentration of sodium^28^. A fitting model for these measurements can be described as:

18 $${\sigma }_{(water-sucrose-sodium)}=\frac{{s}_{1}T0}{1+{({s}_{2}T0)}^{2}},$$

where, s_1_, and s_2_ are coefficients that are computed for each curve of sodium concentration. The fitting model provided a R^2^ of 0.90, 0.98, 0.98, and 0.97 for the sodium concentration of 0.5, 1, 1.5 and 2%, respectively. The coefficient s_1_ exhibit a linear correlation with the sodium concentration that was fit with a R^2^ of 0.99, while the s_2_ was fit to a second order polynomial with R^2^ of 0.99. The plots show that the value of the conductivity lays on the curve, however, the curve is scaled by the concentration of sodium, with the lower concentration of sodium (0.5%) having the lowest curve and 2% sodium being the highest scaled curve. A similar pattern was observed for the water-sucrose-potassium and water-sucrose–sodium-potassium. Another plot that is of significant interest for this work is the relationship between permittivity and conductivity; however, as a function of the T0, as shown in Fig. 5i, where the lower T0, which is approximately 500 ms, has the lower curve, and the largest value of T0 (approximately 1450 ms) has an expanded curve. This plot is of importance because it indicates that the value of the conductivity could be found by selecting the right permittivity and T0, which can be done using machine learning and neural networks algorithms.

## Permittivity estimation

Three types of permittivity estimations are presented in this study based on curve fitting following Eq. (3), and the proposed methods are based on RL and NNF.

### Curve fitting

**Figure 6:**
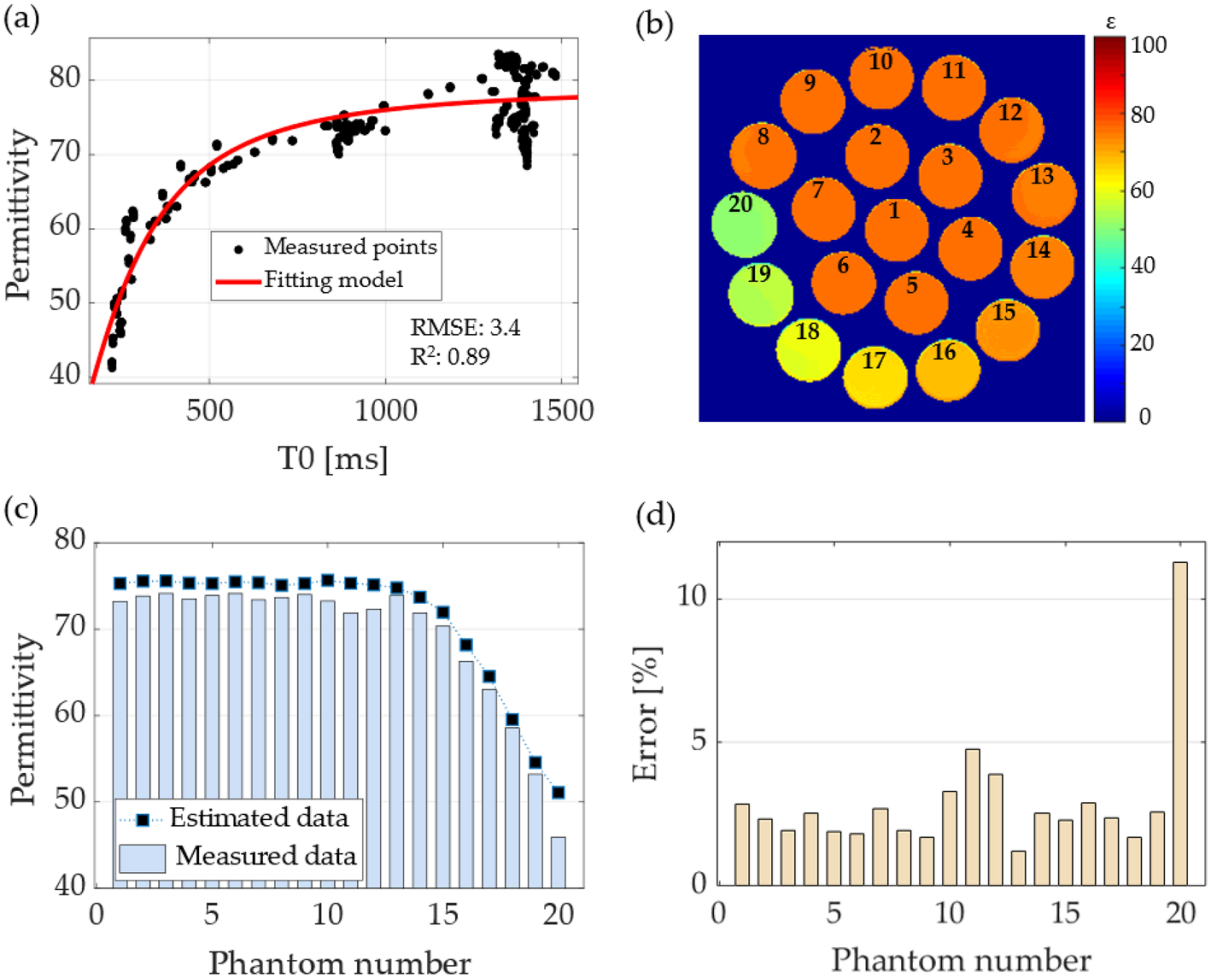
The results of using curve fitting for estimating the permittivity. (**a**) The model and measured points, (**b**) permittivity map for the water-sucrose–sodium-potassium mixture, (**c**) comparison between the measured and estimated data, and (**d**) the percentage of error from the permittivity map estimation.

Following the proposed Eq. (3), all the data were used to determine the corresponding coefficients such that the model could predict the permittivity. The coefficients were found with 95% confidence bound as e1 = 79.01 (78.45, 79.57), e2 = 16.97 (6.10, 27.85), and c = 0.0044 (0.0037, 0.0051). The fit had an RMSE of 3.43 and an R^2^ of 0.89. Figure 6a plots the fitting model and measured data between T0 and permittivity. The permittivity maps were computed by applying the model to the T0 maps shown in Fig. 4. on a pixel-by-pixel basis, respectively. Figure 6b shows the permittivity map computed for the phantoms of the water–sucrose–sodium–potassium mixture. The figure labels the phantom number that correspond to the x-axis of the plots. For comparison with the measured data, the average of the computed permittivity was evaluated for a 5 × 5 pixel ROI at the center of each phantom, as shown in Fig. 6c, where the black squares represent the values of the estimated permittivity using the curve fitting model of Eq. (3), and the bars indicate the measured (target) value of the permittivity. The percentage error was computed for each phantom and plotted in Fig. 6d. The error has an average of 3.6%, and the largest error came from phantom number 20, with an error of 12%.

### Regression learning

We tested 19 different types of RL algorithms with different parameters. Table 1 summarizes the algorithms and their corresponding performance values. The GPR algorithm exhibited the lowest RMSE of 1.90 using an exponential basis, followed by another GPR; however, with a rational quadratic basis. For the same GPR algorithm, the best R^2^ value was 0.96.

**Figure 7:**
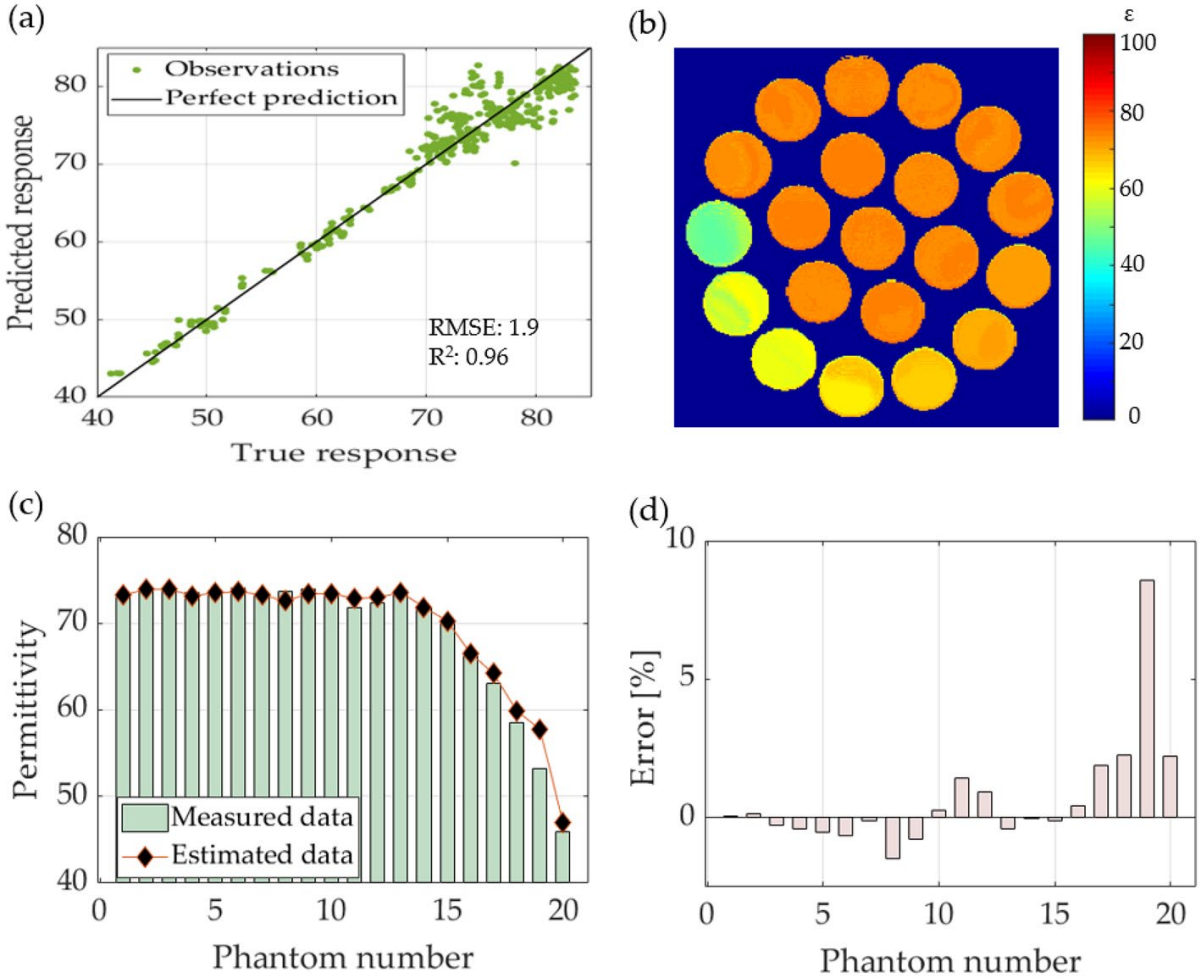
The results of permittivity estimation using regression learning. (**a**) The gaussian regression process model response, (**b**) permittivity map computed with the GPR for the phantoms with a mixture of water-sugar-sodium–potassium, (**c**) comparison between the estimated and measured permittivity for each phantom, and (**d**) error percentage for the computed permittivity for each phantom.

The performances of the exponential GPR model are plotted in Fig. 7a, for the true and predicted responses. This model was used to compute permittivity based on the T0 maps shown in Fig. 4. The computed permittivity map for the phantoms of the water-sucrose–sodium-potassium mixture is shown in Fig. 7b. The same position and size of the ROI as in the curve-fitting results were used to obtain the average value. Figure 7c shows a comparison between the computed permittivity and the measured data, which indicates that the computed values are more accurate than the curve fitting method. Figure 7d shows the percentage error between the computed and measured values. The largest error was 8% for phantom 19, whereas the average error was 0.66%, thereby indicating that the estimation was more accurate than the reference curve fitting model.

### Neural network fit

The performances of the tested NNF methods are summarized in Table 2. The best RMSE and R^2^ during testing of the model were acquired with the Levenberg–Marquardt algorithm of network size 50; however, this model resulted in a non-monotonic function, and consequently, some error values were obtained. The use of a network size of 17 resulted in a monotonic function that provided accurate values.

**Figure 8:**
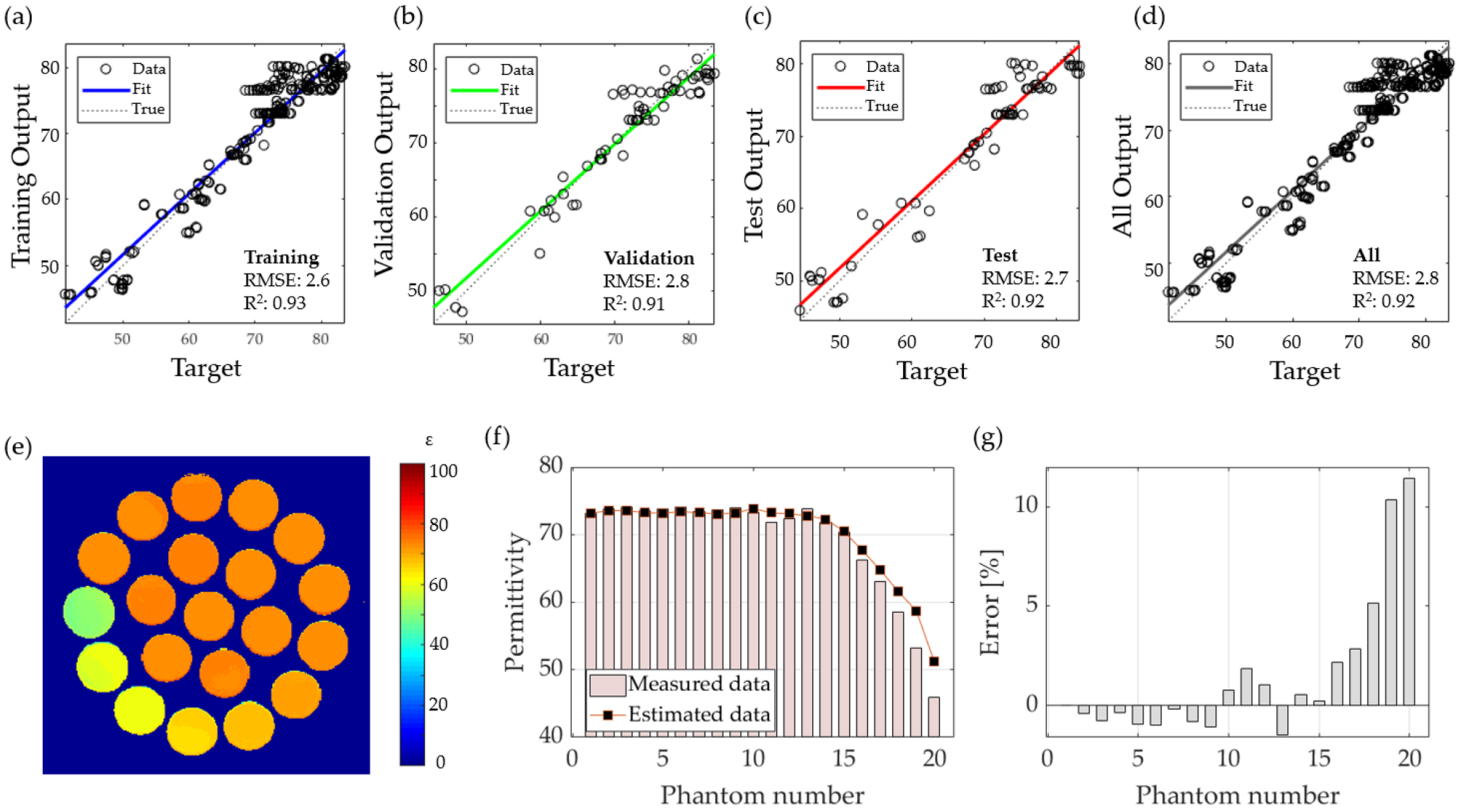
The results of estimating permittivity with neural networks fit, plotting the model performance during (**a**) training, (**b**) validation, (**c**) test and (**d**) everything combined; (**e**) permittivity map computed based on the neural network fit model; (**f**) comparison between the measured and computed permittivity for each phantom; (**g**) percentage error for each phantom.

Based on the tested algorithms, we selected the Levenberg–Marquardt algorithm with a network size of 17, and the training, validation, and test performances are plotted in Figs. 8a–c, respectively. The plot in Fig. 8d demonstrates the training, validation, and test results. Based on the acquired model, we applied to the T0 maps, and permittivity maps were computed for the phantoms of a mixture of water-sucrose–sodium-potassium and the labeling, as shown in Figs. 8e and 6b, respectively. A comparison between the measured data and estimated permittivity is shown in Fig. 8f for each phantom in Fig. 8e. The percentage of error is plotted in Fig. 8g, with a mean error of 1.3%, with the largest error occurring for phantoms 19 and 20, with values of 10% and 11%, respectively. The results indicate that permittivity can be estimated using neural networks with better estimation that the curve fitting, however it exhibits larger error when the values of permittivity are low.

### Analysis of permittivity computation

Comparing the three methods, curve fitting, RL based on GPR, and NNF based on Levenberg–Marquardt, it is observed that the use of the GPR model provided a better estimation of permittivity, with a mean error of 0.66%, whereas the curve fitting resulted in a mean error of 3.6%. The neural network fit also improved the estimation of permittivity, which showed a mean error of 1.3%; however, its performance lies between that of the GPR and CF.

## Conductivity estimation

Similarly, we used three methods to create models to compute conductivity. From Eqs. (6) and (7) and from the correlations described in Fig. 5g,h, the conductivity can be better estimated by combining T0 and permittivity.

### Curve fitting

**Figure 9:**
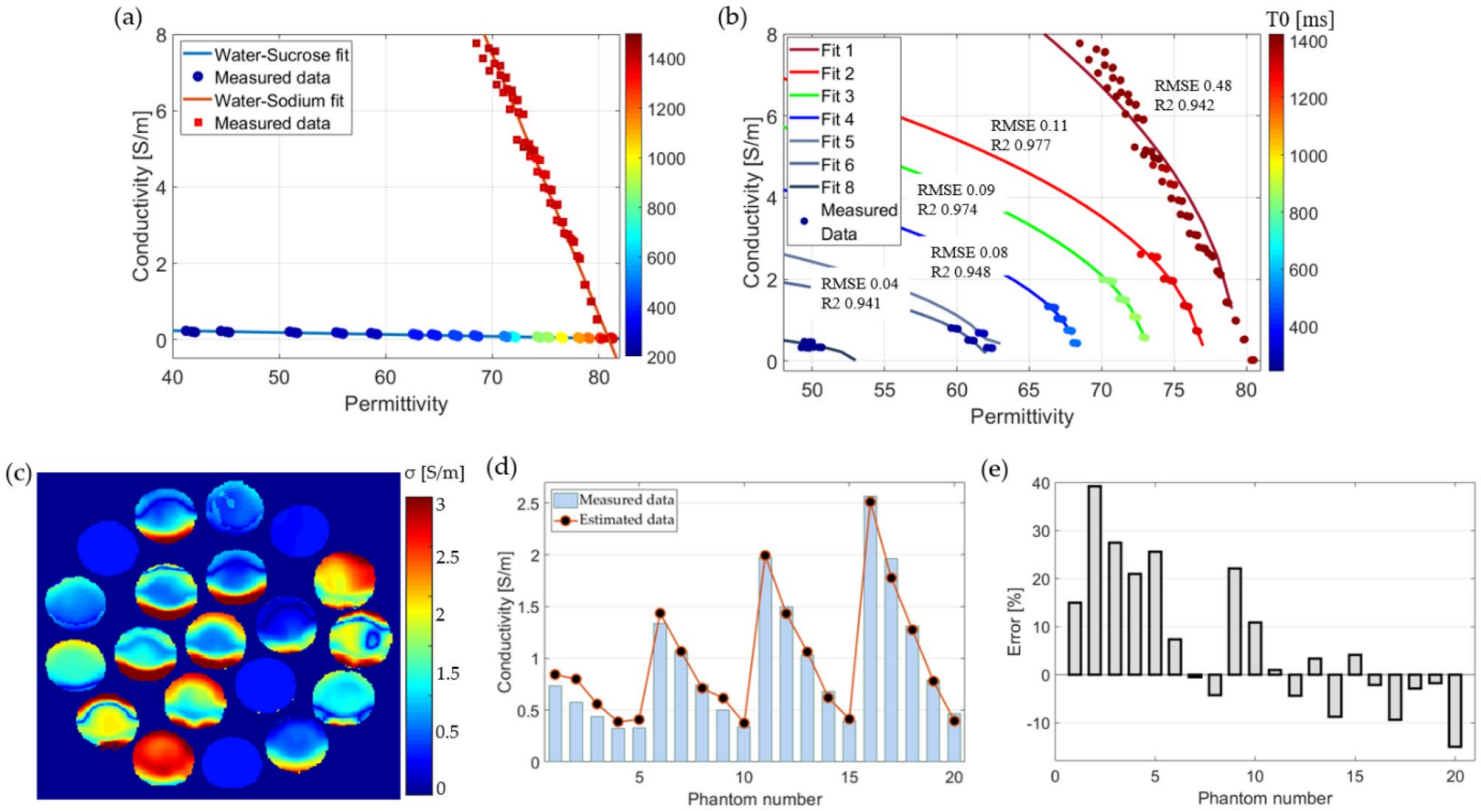
Representation of the relationship between permittivity and conductivity. (**a**) for the mixture of water sucrose and water-sodium with color value assigned to the value of T0, (**b**) curve fitting models based on the T0 value to correlate permittivity and conductivity, (**c**) conductivity map computed with the curve fitting models, (**d**) comparison between the measured conductivity and the computed conductivity, and (**e**) error percentage for the computed conductivity.

The computation of the conductivity using the curve fitting method was performed using Eqs. (6) and (7). The fit models for the phantoms with a mixture of water-sucrose and water-sodium are plotted in Fig. 9a these two plots can be considered as the lower and upper limits for the EPT values according to the ingredients of the phantoms, the plots show the permittivity vs conductivity, but the values have been color mapped to the corresponding T0 value, for a range between 200 to 1500 ms. It can be noticed that the sucrose fit (blue line) has its measured points almost in a horizontal line, with the measured points changing color or T0, indicating the high correlation between T0 and permittivity with the concentration of sucrose. The fit for the mixture of water and sodium indicates that for a similar value of T0, there is a change in permittivity. The model for water-sucrose had an RMSE of 0.013 and R^2^ of 0.94, while the fit for water-sodium had an RMSE and R^2^ of 0.48, and 0.94, respectively. Once these plots were understood, the fit was applied to the other mixtures, and the fit models are shown in Fig. 9b showing the color map variation of T0 and permittivity. The conductivity map was computed using the acquired models, as shown in Fig. 9c. By taking the center ROI for each phantom, a comparison of the measured and computed values is plotted in Fig. 9d, and the error percentage is plotted in Fig. 9e. The mean error percentage was 6% when the ROI was the full size of the phantom, and 4% at the center of each phantom. However, the noise and variation of the permittivity and T0 maps give rise to artifacts, as it can be seen in the Fig. 9c.

### Regression learning

Nineteen RL algorithms were tested to create a model for conductivity estimation based on T0 and permittivity as input variables. Table 3 summarizes the performance of each algorithm. Once again, the GPR model showed the best performance, with an R^2^ of 0.99 and an RMSE of 0.154.

**Table 3:** The performance of regression learning algorithms for conductivity estimation.

Algorithm name	Model type	RMSE	R^2^
Linear regression	Linear	1.29	0.43
Linear regression	Interactions linear	0.99	0.66
Linear regression	Robust linear	1.65	0.07
Linear regression	Stepwise LINEAR	0.99	0.66
Tree	Fine tree	0.37	0.95
Tree	Medium tree	0.58	0.88
Tree	Coarse tree	0.81	0.77
SVM	Linear SVM	1.43	0.30
SVM	Quadratic SVM	0.95	0.69
SVM	Cubic SVM	5.48	0.00
SVM	Fine Gaussian SVM	0.48	0.92
SVM	Medium Gaussian SVM	0.67	0.84
SVM	Coarse Gaussian SVM	1.39	0.34
Ensemble	Boosted trees	0.41	0.94
Ensemble	Bagged trees	0.53	0.90
GPR	Squared exponential GPR	0.27	0.97
GPR	Matern 5/2 GPR	0.25	0.98
GPR	Rational quadratic GPR	0.25	0.98
GPR	Exponential GPR	0.15	0.99

**Figure 10:**
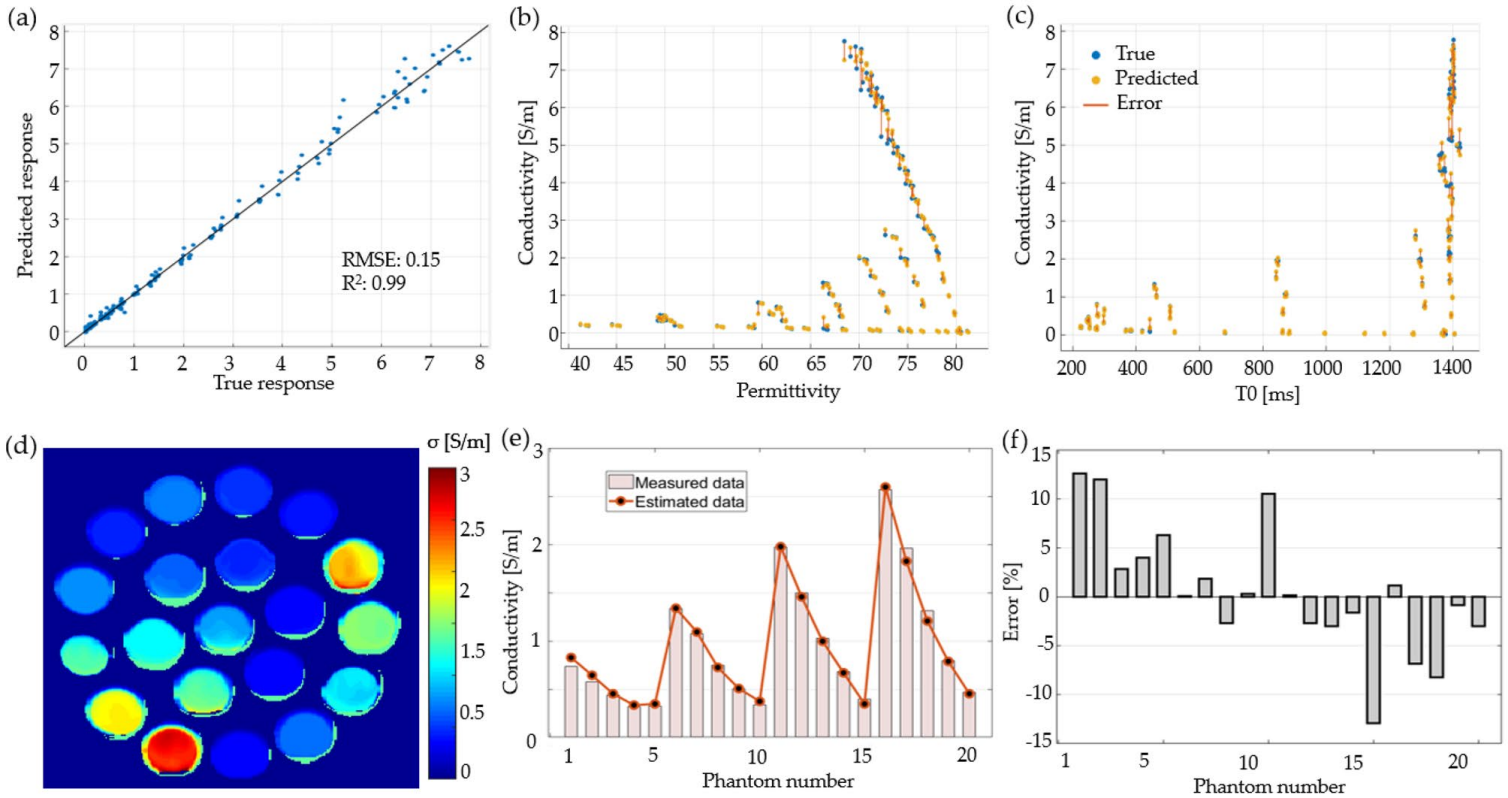
The (**a**) GPR model response for estimating the conductivity. The plot error of the model for the input variable (**b**) permittivity and (**c**) T0. The (**d**) conductivity map computed with the model; (**e**) comparison between the measured conductivity and the computed conductivity; and (**f**) error percentage for each phantom.

Figure 10a shows the performance of the GPR with an exponential basis; the model had an RMSE of 0.15 and R^2^ of 0.99. The responses of the model predictions to permittivity and T0 are plotted in Fig. 10b,c, respectively. These plots show the error between the predicted and measured values. The computed conductivity map using the model is shown in Fig. 10d, and the mean value of the ROI of each phantom is compared with the measured data in the plot of Fig. 10e. Because the model depends on two input variables, the resulting image tends to be noisier. To reduce the effect of noise, we applied a 3 × 3 pixel average before applying the model. The error percentage is plotted in Fig. 10f. The average percentage error was 0.49% for the ROI size of each phantom, with a minimum of -12.87% error in the phantom number fifteen and maximum of 12.6% for phantom number one.

### Neural network fit

**Figure 11:**
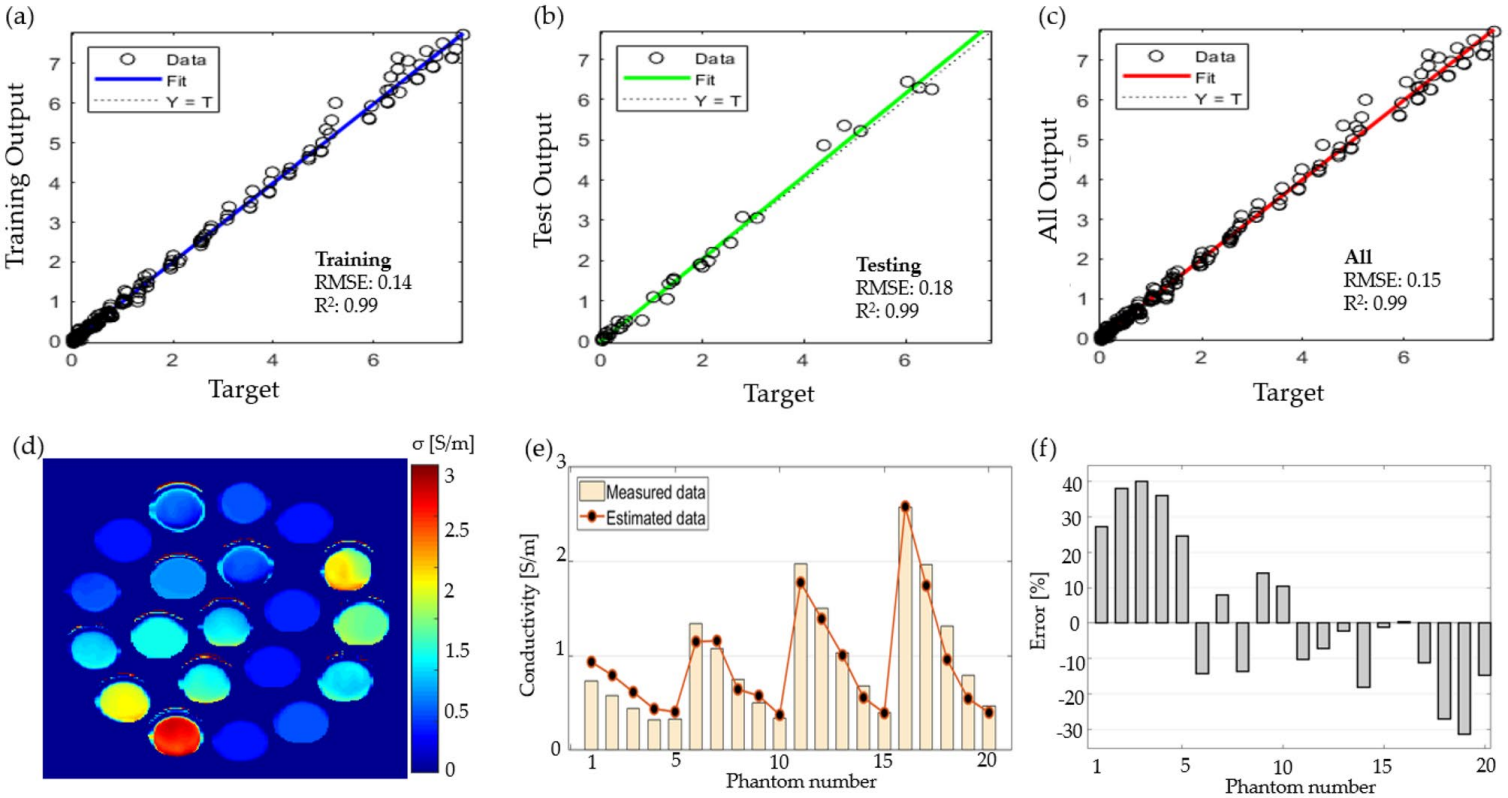
The results using Bayesian regularization neural networks fit, and the plot of the performance of the model during (**a**) training, (**b**) test, and (**c**) everything; (**d**) the computed conductivity map based on the neural network fit; (**e**) comparison between the measured conductivity and the computed conductivity; and (**f**) the error percentage for each phantom.

The fitting results for the different algorithms are summarized in Table 4. As can be seen, the use of Bayesian regularization with a network size of 50 produces an RMSE of 0.15 and R^2^ of 0.99. The performance of the algorithm during training, validation, and testing are shown in Fig. 11a–c, respectively. Conductivity maps reconstructed using the model are shown in Fig. 11d. For this map, we used a 3 × 3 pixel average for the T0 and permittivity maps. Figure 11e shows a comparison between the estimated and measured values for the phantoms. The average percentage of error is plotted in Fig. 11f, and the average error was −2.4%, with a maximum error of 40% for phantom three.

**Table 4:** Performance of the neural fitting algorithms in conductivity estimation.

Algorithm name	Network size	Results	RMSE	R^2^
Levenberg–Marquardt	10	Training	0.42	0.94
Levenberg–Marquardt	10	Validation	0.49	0.91
Levenberg–Marquardt	10	Testing	0.53	0.88
Levenberg–Marquardt	100	Training	0.24	0.98
Levenberg–Marquardt	100	Validation	0.25	0.94
Levenberg–Marquardt	100	Testing	0.4	0.96
Bayesian regularization	50	Training	0.14	0.99
Bayesian regularization	50	Testing	0.18	0.99
Scaled conjugate gradient	100	Training	0.55	0.90
Scaled conjugate gradient	100	Validation	0.50	0.88
Scaled conjugate gradient	100	Testing	0.56	0.88

### Analysis of conductivity estimation.

Conductivity could be estimated using the RL and NNF models; however, contrary to permittivity, conductivity is more susceptible to errors, because of the combination of permittivity and T0 maps that are not homogeneous. For instance, the error of the permittivity estimation was carried over to the conductivity estimation. However, the use of GPR and NNF models improved the computation accuracy of conductivity based on input variables.

## Discussion

This study evaluated the use of RL and NNF for the estimation of the electrical properties of phantoms by measuring the T1 relaxation time using MR images. To validate the acquired results, we created and developed phantoms with different conductivity and permittivity values, which in turn were controlled by mixing different concentrations of water, sucrose, sodium, and potassium. The real values of conductivity and permittivity were measured using an invasive method employing a dielectric assessment kit (DAK), and the experiment was performed for each phantom. The images of each phantom were acquired with a 7 T MRI system using the inversion recovery pulse sequence. The T1 value for each phantom was then computed. Additionally, a scaled value was introduced to improve the uniformity of the T1 estimation, called T0. The plots of T0 vs. permittivity and conductivity showed a correlation of R^2^ 0.9 that can be used to estimate the electrical properties based on the image alone. This work demonstrates that the use of machine learning algorithms can improve the estimation of the permittivity and conductivity.

We presented three methods for computing permittivity and conductivity based on MRI images and T1 values. Compared to curve fitting and neural network fit, the use of regression learning methods can improve the estimation of permittivity. In particular, the neural network fit improved conductivity calculation. The estimation of conductivity is a more complex problem than the estimation of permittivity, since it requires the information of the permittivity and T1 value. Regression learning techniques are better suited for data that follow an equation, whereas neural network fit methods can handle better nonlinear data with complex patterns and relationships.

We believe the study results will be useful for understanding the relationship between EPT and T1 values, such that the use of advanced algorithms can be implemented to further improve EPT computation. The present work was developed with the concept that the sucrose can modify the permittivity, whereas sodium and potassium was used to modify the conductivity. The present work has the limitation that was not tested in vivo tissues. The results obtained in this study have potential clinical applications, as the proposed models can be used to determine the conductivity and permittivity of tissues. The proposed methodology can be expanded to study the properties of tumor tissues and establish a relationship between T1 values of tumor tissues and conductivity. These estimations could be used as biomarkers for diagnosis and monitoring of tumors, and therapies. Furthermore, the models can be expanded to incorporate additional tissue types, which could aid in the characterization and identification of various pathological conditions^53^. The algorithms and proposed equations also have the limitation on the accuracy and image quality of the T1 map acquired, which is also affected by the transmission magnetic field of the radiofrequency coil^54^. Improving the T1 computation could reduce the artifacts and errors in the estimations. The water phantoms that we used in this work have the limitation also that are more susceptible to motion artifacts due to gradient switching, which may be the origin of the artifact in the boundaries of the phantoms, for which agar phantoms could also be used. Susceptibility difference between the empty space and the phantoms could also produce artifacts. One way to reduce this effect is to add a background material to reduce susceptibility artifacts, such as water or agar. This work and concept could be expanded to include other neural networks algorithms and methods.

Experimental data is provided in the supplementary material, such that new algorithms can be developed. A niche application of the models and data could be to establish a normalization for the reference phantoms used for Sodium imaging. In which the Sodium concentration, T1, permittivity and conductivity should be considered so that the experiments and methods can be validated.

## Supplementary Information

Supplementary Information.

## Data Availability

The datasets used and/or analyzed during the current study are available from the corresponding author upon reasonable request.

## References

[1] Sindhu, T. S., Kumaratharan, N. & Anandan, P. A review of magnetic resonance imaging and its clinical applications. In *2022 6th International Conference on Devices, Circuits and Systems (ICDCS)* 38–42. (IEEE, 2022).

[2] Roh K, Kang H, Kim I (2014). Clinical applications of neuroimaging with susceptibility weighted imaging. J. Korean Soc. Magn. Reson. Med..

[3] Sperling R (2011). The potential of functional MRI as a biomarker in early Alzheimer's disease. Neurobiol. Aging.

[4] Dhole NV, Dixit VV (2022). Review of brain tumor detection from MRI images with hybrid approaches. Multimed. Tools. Appl..

[5] Thompson SM (2021). Body interventional MRI for diagnostic and interventional radiologists: Current practice and future prospects. Radiographics.

[6] Bradley WG (1993). MR appearance of hemorrhage in the brain. Radiology.

[7] Baur A (2020). Evaluation of T1 relaxation time in prostate cancer and benign prostate tissue using a modified look-locker inversion recovery sequence. Sci. Rep..

[8] Wagner-Manslau C, Lukas P, Herzog M, Kau R, Beckers K (1994). MRI and proton-NMR relaxation times in diagnosis and therapeutic monitoring of squamous cell carcinoma. Eur. Radiol..

[9] Gabriel G (2004). Measurements of T1 and T2 relaxation times of colon cancer metastases in rat liver at 7 T. MAGMA.

[10] Pettersson HO (1988). Musculoskeletal tumors: T1 and T2 relaxation times. Radiology.

[11] Gabriel S, Lau RW, Gabriel C (1996). The dielectric properties of biological tissues: II. Measurements in the frequency range 10 Hz to 20 GHz. Phys. Med. Biol. Phys. Med. Biol..

[12] Matković A, Kordić A, Jakovčević A, Šarolić A (2022). Complex permittivity of ex-vivo human, bovine and porcine brain tissues in the microwave frequency range. Diagnostics.

[13] Gabriel, C. Compilation of the dielectric properties of body tissues at RF and microwave frequencies. King's Coll London (United Kingdom) Dept of Physics (1996).

[14] Leijsen R, Brink W, van den Berg C, Webb A, Remis R (2021). Electrical properties tomography: A methodological review. Diagnostics.

[15] Katscher U, van den Berg CA (2017). Electric properties tomography: Biochemical, physical and technical background, evaluation and clinical applications. NMR Biomed..

[16] Liu J, Wang Y, Katscher U, He B (2017). Electrical properties tomography based on B1 maps in MRI: Principles, applications, and challenges. TBME.

[17] Van Lier AL (2014). Electrical properties tomography in the human brain at 15, 3, and 7T: A comparison study. Magn. Reson. Med..

[18] Lesbats C (2021). High-frequency electrical properties tomography at 9.4 T as a novel contrast mechanism for brain tumors. Magn. Reson. Med..

[19] Bulumulla SB, Lee SK, Yeo DTB (2012). Conductivity and permittivity imaging at 3.0 T. Concepts Magn. Reson. Part B Magn. Reson. Eng..

[20] Gurler N, Ider YZ (2017). Gradient-based electrical conductivity imaging using MR phase. Magn. Reson. Med..

[21] Michel E, Hernandez D, Cho MH, Lee SY (2014). Denoising of B 1+ field maps for noise-robust image reconstruction in electrical properties tomography. Med. Phys..

[22] Liu C, Jin J, Guo L, Li M, Tesiram Y, Chen H, Liu F, Xin X, Crozier S (2018). MR-based electrical property tomography using a modified finite difference scheme. Phys. Med. Biol..

[23] Michel E, Hernandez D, Lee SY (2017). Electrical conductivity and permittivity maps of brain tissues derived from water content based on T1-weighted acquisition. Magn. Reson. Med..

[24] Markel VA (2016). Introduction to the Maxwell Garnett approximation: Tutorial. JOSA A.

[25] Shah NJ, Abbas Z, Ridder D, Zimmermann M, Oros-Peusquens AM (2022). A novel MRI-based quantitative water content atlas of the human brain. Neuroimage.

[26] Shah, N. J., Ermer, V. & Oros-Peusquens, A. M. Measuring the absolute water content of the brain using quantitative MRI. In Magnetic Resonance Neuroimaging 29–64 (2011). Humana Press. 10.1007/978-1-61737-992-5_3 21279597

[27] Watanabe T, Wang X, Tan Z, Frahm J (2019). Magnetic resonance imaging of brain cell water. Sci. Rep..

[28] Hernandez, D. & Kim, K.N. Correlation analysis between the complex electrical permittivity and relaxation time of tissue mimicking phantoms in 7T MRI (2022). 10.1038/s41598-022-19832-y PMC9474530 36104392

[29] Raju, G. G. Dielectrics in electric fields: Tables, Atoms, and Molecules. CRC press (2017).

[30] Pethig R (1985). Dielectric and electrical properties of biological materials. J. Bioelectr..

[31] Schilling KG, Landman BA (2019). AI in MRI: A case for grassroots deep learning. Magn. Reson. Imaging.

[32] Zhao, R., Zhang, Y., Yaman, B., Lungren, M.P. & Hansen, M.S. End-to-end AI-based MRI reconstruction and lesion detection pipeline for evaluation of deep learning image reconstruction. arXiv preprint arXiv:2109.11524 (2021).

[33] Hötker AM, Da Mutten R, Tiessen A, Konukoglu E, Donati OF (2021). Improving workflow in prostate MRI: AI-based decision-making on biparametric or multiparametric MRI. Insights Imaging.

[34] Das, S., Nayak, G.K., Saba, L., Kalra, M., Suri, J.S. & Saxena, S. An artificial intelligence framework and its bias for brain tumor segmentation: A narrative review. *Comput. Biol. Med.* 105273 (2022). 10.1016/j.compbiomed.2022.105273 35228172

[35] Chen Y (2022). AI-based reconstruction for fast MRI—A systematic review and meta-analysis. Proc. IEEE.

[36] Hampe, N., Katscher, U., van den Berg, C.A., Tha, K.K. & Mandija, S. Deep learning brain conductivity mapping using a patch-based 3D U-net. arXiv preprint arXiv:1908.04118 (2019). 10.1088/1361-6560/ab9356 32408291

[37] Gavazzi S (2020). Deep learning-based reconstruction of in vivo pelvis conductivity with a 3D patch-based convolutional neural network trained on simulated MR data. Magn. Reson. Med..

[38] Sajib SZ, Chauhan M, Kwon OI, Sadleir RJ (2021). Magnetic-resonance-based measurement of electromagnetic fields and conductivity in vivo using single current administration—A machine learning approach. PLoS ONE.

[39] Leijsen R, van den Berg C, Webb A, Remis R, Mandija S (2022). Combining deep learning and 3D contrast source inversion in MR-based electrical properties tomography. NMR Biomed..

[40] Stulp F, Sigaud O (2015). Many regression algorithms, one unified model: A review. Neural. Netw..

[41] Krämer, W. & Sonnberger, H. The linear regression model under test. Springer Science & Business Media (2012).

[42] Morgan, J. Classification and regression tree analysis. Boston: Boston University, 298 (2014).

[43] Noble WS (2006). What is a support vector machine?. Nat. Biotechnol..

[44] Shi, J. Q. & Choi, T. Gaussian process regression analysis for functional data. CRC press (2011).

[45] Tong Y, Yu L, Li S, Liu J, Qin H, Li W (2021). Polynomial fitting algorithm based on neural network. TPRIS.

[46] Rodgers CT, Piechnik SK, DelaBarre LJ, Van de Moortele PF, Snyder CJ, Neubauer S, Vaughan JT (2013). Inversion recovery at 7 T in the human myocardium: Measurement of T1, inversion efficiency and B1+. Mag. Reson. Med..

[47] Marquardt DW (1963). An algorithm for least-squares estimation of nonlinear parameters. SIAM J. Appl. Math..

[48] Hagan MT, Menhaj MB (1994). Training feedforward networks with the Marquardt algorithm. IEEE Trans. Neural Netw..

[49] Hagan MT, Demuth HB, Beale MH (1996). Neural Network Design, Boston.

[50] Møller MF (1993). A scaled conjugate gradient algorithm for fast supervised learning. Neural Netw.

[51] MacKay DJ (1992). Bayesian interpolation. Neural Comput.

[52] Van Lier, A.L.H.M.W., de Bruin, P.W. & Aussenhofer, S.A. 23Na-MRI and EPT: Are sodium concentration and electrical conductivity at 298 MHz (7T) related?. *In Proc Intl Soc Mag Reson Med.* **21**, 115 (2013).

[53] Tha KK, Katscher U, Yamaguchi S, Stehning C, Terasaka S, Fujima N, Shirato H (2018). Noninvasive electrical conductivity measurement by MRI: A test of its validity and the electrical conductivity characteristics of glioma. Eur. Radio..

[54] Hernandez D, Kim KN (2020). A review on the RF coil designs and trends for ultra high field magnetic resonance imaging. iMRI.

